# Editorial: An evolving challenge: pathogenicity, control and elimination of the evolving PRRSV and PCV threat

**DOI:** 10.3389/fvets.2026.1938653

**Published:** 2026-08-04

**Authors:** Osamu Taira, Chaosi Li

**Affiliations:** 1 Nippon Institute for Biological Science, Tokyo, Japan; 2 Elanco Animal Health, Shanghai, China

**Keywords:** diagnostics, disease control, molecular epidemiology, porcine circovirus, porcine reproductive and respiratory syndrome virus, surveillance, vaccination, viral evolution

Porcine reproductive and respiratory syndrome virus (PRRSV) and porcine circoviruses (PCVs) remain among the most consequential pathogens affecting modern swine production. Their impact is sustained not only by reproductive failure, respiratory disease, and production losses, but also by their capacity for genetic change, immune modulation, persistent circulation, and adaptation to complex production systems. Vaccination, molecular diagnostics, and biosecurity have all improved disease control, yet the continued emergence of new variants reminds us that successful strategies cannot remain static. This Research Topic brings together six contributions that reflect this reality, moving from viral evolution and molecular mechanisms to practical surveillance and vaccine development.

Understanding how these viruses change is the starting point for rational control. Zhang et al. characterized the genetic diversity and pathogenicity of contemporary PRRSV-2 strains circulating in China. Their work highlights recombination as a major force shaping PRRSV diversity and shows why sequence data are most informative when interpreted alongside virological and pathogenicity findings. A newly detected strain matters not simply because it occupies a distinct position on a phylogenetic tree, but because of what it can do in the host: replicate, spread, evade immunity, and cause disease.

At the molecular level, Zheng et al. reviewed the many functions of nonstructural protein 2 (NSP2), one of the most variable proteins in the PRRSV genome. NSP2 contributes to viral replication and interacts with host regulatory and immune pathways, while its extensive variability mirrors the broader evolutionary plasticity of PRRSV. Read together, these two contributions link population-level viral evolution with the molecular processes that support replication, adaptation, and immune evasion. They also underscore a point that is easy to overlook: genetic variation becomes meaningful only when its biological consequences are understood.

As viruses evolve, surveillance must become more practical, more representative, and better suited to decisions made at herd level. Plut et al. evaluated oral fluid both as a non-invasive matrix for PRRS monitoring and as part of a controlled-exposure strategy during gilt acclimatization. Their findings show that oral fluid can provide a practical window into group-level infection dynamics, while also making clear that contaminated ropes alone are not a reliable substitute for effective exposure. This balance between promise and limitation is important. Diagnostic tools should be judged not only by analytical performance, but also by how they behave in the realities of commercial production.

Turlewicz-Podbielska et al. extended the same field-oriented logic to PCV2 surveillance by evaluating testicular processing fluid for the detection of viral DNA and virus-specific antibodies. Because this material is already generated during routine procedures, it offers a way to gather herd-level information without additional invasive sampling. The study also shows that matrix-specific cut-offs and careful attention to pooling are essential, particularly when low-level positives may otherwise be missed. Together, these two studies illustrate that innovation in diagnostics is not limited to inventing new assays; it also lies in making better use of specimens that farms already produce.

Vaccination remains central to PRRSV and PCV control, but viral diversity means that vaccine performance must be revisited continually. Suh et al. assessed the reproductive protection conferred by a modified-live PRRSV vaccine against a heterologous Korean PRRSV-2 challenge in pregnant sows. Their study is especially valuable because reproductive disease is among the most damaging consequences of PRRS, and protection in breeding animals cannot be fully inferred from experiments in growing pigs. The observed improvements in reproductive outcome, virological measures, and cellular immune responses provide practical evidence for cross-protection in a biologically and economically relevant model.

Liu et al. explored a different path by developing a baculovirus-engineered bivalent subunit vaccine candidate targeting the capsid antigens of PCV2 and PCV3. The study addresses an increasingly important need: broadening protection as more than one porcine circovirus contributes to field disease. The candidate induced humoral and cellular immune responses and reduced pathological damage following challenge, supporting further development. As with any new vaccine platform, field validation will be crucial, but the work offers a clear example of how antigen design can be adapted to an evolving epidemiological landscape.

Taken together, the six articles present PRRSV and PCV control as a connected process rather than a collection of separate tasks. Genomic surveillance identifies emerging diversity; molecular studies help explain its biological significance; practical sampling strategies convert surveillance into herd-level information; and vaccine studies test whether existing or newly designed tools remain fit for contemporary viruses. Progress in one area strengthens the others, and weaknesses in one can limit the whole system.

Elimination remains an ambitious goal, particularly where multiple viral lineages circulate through interconnected production networks. Even so, more durable control is achievable when molecular epidemiology, functional virology, diagnostics, vaccination, biosecurity, and farm management are integrated rather than applied in isolation. Continued collaboration across countries, disciplines, and the swine industry will be essential, as will timely sharing of genomic, experimental, and field data. The message of this Research Topic is therefore both simple and demanding: as PRRSV and PCVs continue to evolve, the ways in which we understand, monitor, prevent, and control them must evolve as well.

